# Extraction of Teeth

**Published:** 1853-07

**Authors:** J. Taylor


*Extraction of Teeth.* By Dr. J. TAYLOR. [Continued.]

In our last, we gave a general discription of the forceps, used in extracting the teeth of the superior maxilla. They consist of first, an anterior and posterior molar forcep, for either side—making four pairs. Second, a dens sapientiæ forcep, and third a biscuspid forcep. To these we prefer to add a small forcep adapted to the lateral incisor teeth, and a pair for either side of molar forceps, the outer beak with one point so as to pass between the labial roots of these teeth. When thus perfected we have nine pairs. For ordinary use we may reject all but the two pairs of molars, one dens sapientiæ, one biscuspid; which applies, also, to the cuspidati and incisors, making but four pairs.

For the teeth of the inferior maxilla, we have first right and left molars. The right, which is that which applies to the teeth of the right side, is really the hawk's bill forcep, one blade being much longer than the other passing over the crown of the teeth, and applying to their palital face. This instrument is slightly curved in the bar, so that it will lay hold of the posterior molar with facility. One blade of the handle curves around the little finger when in the operators hand. The beaks are grooved to suit the two roots of these teeth, the central point being the longest, and passing between the roots. The blades of these forceps should be grooved out to fit the crowns of these teeth, and made smooth on their inner surface, so that in being applied when the points of the beaks have enclosed the crown of the tooth above the body, they will slip far up on the neck of the tooth, as far indeed as the alveolus will permit. This adaptation may be such, that when the roots are strait, the mere closing of the forcep on the tooth will raise it from the socket. This description or adaptation to the teeth, will suit for both right and left. The form however for the forcep of the left side is entirely different; it opens reversely from that of the right, this forcep has two curves one between the beak and joint, which permits it to pass back over the anterior teeth, and apply to the posterior molar, or even the dens sapientiæ. The second curve is near the joint, between this and where the handles are turned to fit the hand. These curves enable the operator, to pass the forceps obliquely in the mouth from the right side, and lay hold of the tooth on a line with the direction of the alveolus. The adaptation, indeed, in this respect, is far better than the molar for the right side, excepting, indeed, where we have as is sometimes the case, the anterior molar leaning a little out of the circle in which the other teeth are set, that, is not pointing to the center of the alveolar arch. It will be recollected, that the position for the extraction of these teeth, is a little to the right, and back of the patient, with the chin embraced in the left hand of the operator. We regard this forcep to us, as one of the most available in the whole set, and its construction, gave us more trouble than any improvement we have made in extracting instruments.

We think that we hadnot less than half a dozen pairs made, before we could get the curve and the adaptation which we wanted. After having thoroughly tested its utility, we had a pair made for the biscupides of the same side, made after the same general pattern; the only difference, the blades being adapted to these teeth. With these forceps the force can be applied on a line with the direction of the socket, which we regard as of great importance, and the forcep in general use for the teeth of the right side, will not permit this excepting where a tooth stands pointing without the circle.

For the biscuspides of the right side, we have a hawk's bill forcep which we use also on the cuspidati of the same side. For the incisors, we use the strait or curved root forcep.

The dens sapientiæ below we have formerly regarded as one of the most difficult teeth to remove. We are glad to say, that an instrument has been brought into use for their removal, which has done away with, in almost all cases, the difficulty. This is, what is called Physic's elevator. It is so much like a forcep in every thing but the blades; that we shall now give it a place with these valuable instruments.

This instrument is especially adapted to the dens sapientiæ of the lower jaw, yet we often use it for the upper. The blades are bent, at or near a right angle with the bar of the instrument. The blades are made varying from half an inch, to an inch in length. Made convex on one side, and concave on the other, and are designed to pass between the posterior molar and dens sapientiæ teeth. The convex surface when applied sliding over the posterior face of the molar teeth, catching the dens sæpientia in their concave surface, and lifting the tooth from its socket, on a line somewhat with the curve of the roots. We have never seen any other instrument except an elevator of some kind, which would do this

No forcep or turnkey can be applied to effect their removal in this manner. There are some one or two conditions of the teeth, where the application of this instrument is inadmissible. First, when the contiguous molar is gone, and second, when it is so much decayed that it presents no surface, as a fulcrum for the blades of the instrument, or where the anterior approximal face of the dens sapientiæ, is decayed so much that there is no point above the edge of the alveolus, for the point of the instrument to play upon. The molar may be loose, and would not with safety bear the pressure. We would, however, here remark, that the general apprehension of danger to this tooth, in the application of this instrument, is by no means well founded. The construction of the instrument is such that the force is exerted almost entirely on the teeth to be removed. A little sleight, however, in the application of this instrument, faciliates very much, the operation. The head should be held firm, and as the instrument is closed, forcing the blades between the teeth, pressure is made backwards against the dens sapientiæ, undue pressure will thus be prevented against the molar. The application of force, here raises the tooth partially out of the socket, throwing it slightly backwards. It is then removed with a small pair of forceps. We will give a recent case showing the application of this instrument—a few days since, a French-man applied, to have the left dens sapientiæ of the inferior maxilla removed. The tooth had been a source of pain for near a year, and the swelling and induration extended from the ear along the lower jaw, to near the sympasis, and down the neck some five or six inches. When he first called, the mouth could not be opened more than three fourths of an inch, between the incisor teeth. After using force with a wedge, for two or three days, about an inch of space was obtained. The swelling was so great that we could not apply our old common shaped elevator which has but a slight curve in the bar, and we could not pass what is called Physic's Elevator, made in the bar about as thick as a common forcep, back to the tooth. While pressure was being applied to gain a wider opening of the mouth, we had an elevator made with the bar sufficiently curved to allow the point to be passed back between the teeth, but the tooth was so sore, and the patient so unsteady, that we could not apply this instrument with that security and precission we desired. We then took an old pair of elevating forceps, which in the bar is not more than half as thick as usual, and then filed off and rounded down the points of the blades, so that by using some force, we passed it back to the tooth, having first (to gain all the space and room, for the instrument possible) opened the blades and thus slipped it over and back of the posterior molar. So soon as we felt the point of the blades of the instrument pass back on to the posterior level of the crown of the molar, we commenced closing the instrument and forcing it downwards, so that the points of the blades should get as low down as the imbedded dens sapientiæ tooth. This movement caught the tooth, and raised it up nearly on a level with the molar, when with a crooked bicuspid-forcep, the tooth was removed from the mouth. The upper portion of the crown of this tooth pressed against the posterior face of the molar, and did not come up, on a level with the largest circumference of the crown of the molar.

The roots curved back under the coronoid process of the bone, and no forcep in this condition of the mouth even if in a healthy state, could have been brought to bear on the tooth, and had this been possible the force for the extraction of the tooth would have been applied against the resistance of the molar and the crooked roots of the dens sapientiæ. We do not give this, as specially an extraordinary case, only so far as the extensive swelling was concerned, and the difficulties occasioned from this cause in the removal of the tooth. Here had it not been for an old instrument of my brother Joseph Taylor, formerly of Maysville, Kentucky; which he had made while at that place, for first separating roots, and then for the removal of such teeth, we should have had in all probability much more difficulty than we had.

This instrument, indeed, was made and in use years before the physic's elevators were seen or known of in the West. He used it for two or three years before any were in use to my knowledge, and that long before we could consent to try it, or have one made for ourself. Dr. Griffith, of Louisville, has an old instrument, which he uses to great advantage for a similar purpose, and is just the same in principle, only the blades open the reverse just like a hawk's bill forcep. We have used this instrument, the elevator, for loosening the posterior molar when the dens sæpientia was out, and the anterior molar good and fine, the posterior molar being decayed below the gum ; only, a firm portion resting against the anterior molar, and in no case have we ever injured in the least any tooth which we have used as a fulcrum, in the application of this instrument.

We have now given a general idea of the forceps we use in the extraction of teeth; not, it is true, having described the root forceps. These we shall merely remark consist, with us, of some three or four pairs made very sharp at the point of the blades, and so tempered as not to break by the application of considerable force. We have them of various curves from nearly strait to one at near right angles—with us a great desideratum is adaptation to the root. The blades should encircle the roots neatly and accurately, allowing the pressure in their application to be as equable on the frail root as possible. They should be so made on their grooved points, that they will slip up upon the roots to the edge of the alveolus, without any difficulty, and so sharp at the points that they will separate the gum as they pass up, and if need be, can be forced into the socket itself.

We shall give the application of these instruments somewhat in the order in which we have described them—remarking, however, that we have already been, perhaps, sufficiently explicit in relation to the elevator—as we progress in this description it will be necessary to allude to the general anatomical appearance of the different teeth, and sometimes their relation to the parts with which they are connected, and as we are writing more for the beginner than the expert operator, we shall as far as we can, give the difficulties in the way. We do not always get out a tooth the first effort, and we have very little patience with those who say they do.

We have first the molars of the inferior maxilla of the right side, and for their removal a hawk's bill forcep, the beak for the palital face of the tooth being long, reaching over the crown of the tooth, and adjusting the point of the blade, just where the two roots of these teeth begin to separate. The short beak at the same time takes its place similarly on the labial face of the teeth. We are now standing a little to the right and behind our patient on a stool, and have hold of the chin with our left hand. If the tooth leans inward, the bar of the instrument comes almost in contact with the upper teeth, and hence very little force can be exerted inward for loosening the tooth. If an anterior molar the roots often separate, so a line drawn around them, will measure more than around the crown of the tooth. The roots generally are much flattened, transversely, and turn a little backwards.

The first application of force we make, is upward and outward, from the fact that the inward motion is not often to any extent admissible. We have, it is true, here to contend with the resistance of the outer plate of the inferior maxilla, and often a firm and unyielding alveolus. This first motion, however, generally loosens the tooth, and if we feel it still confined in the socket, we move inward and backward, and then outward. These motions, where the roots separate much, is often necessary, and in the anterior molar this is often the case, but for the second molar the outward and upward motion is generally sufficient. So far, this is all easy, but say the tooth is frail, and the first motion it breaks off below the gum. If the tooth is anything like sound at the neck, we would not, however, anticipate the breaking of the tooth. And if after a careful examination of the teeth, we found there would be danger of breaking, we should, in applying the forcep, force it as low on the tooth as possible, and as it was pressed to its place, give a short rocking motion inward and outward, loosening if possible the tooth in this way, before the direct force for extraction is applied.

We would here remark that in all cases where much decay is manifest, we always insist on carefully examining the extent of the decay, also, ascertain the condition of the alveolus, ascertain if it is firm and strong on the upper margin, and if it adheres closely to the tooth, and comes up high on the neck—see with my probe if the decay dips below the border of the alveolus. The neck of the teeth, and the condition of the alveolus, can be examined with a gum lancet which I have made flat on one side and rounded on the other, and rather press the gum away, than cut it. The examination of the depth of decay can generally be well made, without touching an exposed nerve. These are points where teeth are much decayed, that are very essential and if properly attended to, a vast amount of suffering may be often avoided.

But we have now a broken anterior molar of the lower jaw to extract. The first thing to be done, is to examine carefully with a gum lancet, the condition of the roots. See if there be any point which will bear the application of the instrument. If the decay has been principally on the posterior part of the tooth it may have broken very low at this part, and anteriorly not so low, but that a root forcep will take hold of the anterior root. If then this stands up a little above the alveolus, we take the gum lancet, and separate the gum where the forcep blades apply thoroughly to the alveolus, and often force the lancet into the socket on either side, this enables us to force the forcep as far as possible down on to the firm portion of the root, so that another break of the tooth will not take place. In this operation we would expect to remove the anterior root and often if the union is strong, both, if not the posterior root will be left in. If the root is exceedingly frail, the forcep we use in this part of the operation, is the most curved root forcep we have, and we stand rather to the right and front of the patient, holding the chin with our left hand, and as we force the forcep down as low as possible, we make short motions inward and outward, taking care not to close the forcep so tight as to break the root, or prevent its slipping down on the root. Fear of hurting the patient, or indecision only adds to the difficulty, and often occasions twice as much pain to the patient. A firm hand with the operator, and firm head with the patient, will almost invariably insure success.

The posterior root is now to be removed, and a careful examination should be made, and if there be a point within and without sufficient for the application of the forcep, we apply as in the other root. In most of these cases where the root is frail and the alveolus can be forced a little open we do this before applying our focep. In this operation we have two objects in view; first, loosening of the root, and second, a more secure application of the forcep. The opening of the alveolus, is usually not attended with much pain, for we are now operating on almost entirely osseous structure. The ininstrument we use for this purpose, is a sharp pointed and strong gum lancet, made as before remarked, a little concave on one side, and convex on the other, that is something like the point of a root forcep, only sharper. As this is forced in by the root, the socket must open or the root raise up, for there is not room for both. We have a pair of these, besides a strait one for the upper jaw. But we may now be told, that teeth sometimes break so low down, as to forbid the application of the forcep as described. This is very true, and we will now state a case. The tooth breaks on a level with the upper margin of the alveolar processes, and this is firm and unyielding, so much so, that it is not practicable to open the socket as decribed. We then proceed as follows: We take our lancet, and pierce the gum abuut two lines below the upper margin of the alveolus, and make from this point which strikes between the roots a circular incission upward and forward and also backward, as far as the center of either root and indeed to in a measure embrace both roots, one cut with the lancet either way effects this. We then take a beveled edge, cutting instrument, such as we use for cutting down the enamel over decay. With this we cut the bone as before we had cut the gum. We then pass in our gum lancet, and by a little force separate this portion of the alveolus from the roots, and remove it out of the way. We have now free access to the labial portion of the roots.

We have already described a hawk's bill forcep with a single pointed beak or point. This is for these cases. We separate now the gum slightly from the alveolus on the palital side; we now apply our forcep with one point, between the roots on the outside and embracing a little of the alveolus within, the first motion is now made inward and upward, and difficult indeed must be the case, if the roots are not either removed, or separated and loosened by this operation. I believe we have never had to cut away the process of the palital side of the roots. We have a few times forced open the socket between the roots, and got room for the point of our forcep blade, and prefer doing this when it is easily done. If the roots separate and do not come entirely out, they generally are more easily removed with the root forcep.

We have confined our remarks particularly to the operation of removing the anterior inferior molar of the right side, but it is equally applicable to the posterior molar of the same side. The roots in the latter are more frequently united, and generally require less force for removal. For the molar of the left side, (inferior maxilla,) our forcep is applied obliquely across the mouth, our position is behind and a little to the right of the patient, and as before, we hold the chin with our left hand. The construction of this forcep gives us opportunity to make freely the inward motion in the loosening of this tooth, and hence we find the teeth of this side more easily removed. The application of force is on these teeth first inward then upward and outward. If the tooth holds in the socket after these movements have been made, we direct the force backward and upwards; rocking it so as to disengage it from the alveolus. These the anterior molars of the inferior maxilla, are sometimes held in after having been perfectly loosened, requiring after this considerable force for their removal. This is owing to the diverging and crooked condition of the roots, most frequently curving backwards. We had a case a few years since of this kind; where after the teeth had been raised two or three lines, we could not with all the force we felt willing to apply, remove the tooth with the forcep. We divided the crown with a separating file, and separating forcep, and took each root out separately, and when put together, it was apparent that roots or processes one must have broken but for this separation.

